# Performance and intestinal health of piglets in the nursery phase subjected to diets with condensed black wattle (*Acacia mearnsii*) tannin

**DOI:** 10.5713/ab.24.0112

**Published:** 2024-08-23

**Authors:** Kelly Lais de Souza, Cleandro Pazinato Dias, Marco Aurélio Callegari, André Friderichs, Alcides Oliver Sencio Paes, Rafael Humberto de Carvalho, Caio Abércio da Silva

**Affiliations:** 1 Animal Science Program, Center of Agrarian Sciences, State University of Londrina, Londrina, PR 86057970, Brazil; 2 Akei Animal Research, Fartura, SP 18870970, Brazil; 3 Tanac S.A, Timbaúva, Montenegro, RS 95780-000, Brazil

**Keywords:** Additives, Digestibility, Intestinal Microbiota, Sodium Butyrate, Tannin

## Abstract

**Objective:**

The objective of this study was to evaluate the use of condensed tannin from black acacia (*Acacia mearnsii*) as a substitute additive for zinc oxide and growth-promoting antibiotics on the performance, digestibility, and intestinal health of piglets in the nursery phase.

**Methods:**

A total of 200 PIC piglets that were 22 days old and weighed 6.0±0.9 kg were subjected to four treatments in the nursery phase (22 to 64 days of age): CONTR (control diet); ENR+ZnO (control diet + 10 mg/kg of enramycin + 2,500 mg/kg of zinc oxide during the first 21 days); BUT (control diet + 900 mg/kg of sodium butyrate) and TAN (control diet + 2,000 mg/kg of condensed tannin). The experimental design was a randomized block with 4 treatments and 10 replicates, with a pen of five animals each as the experimental unit. The zootechnical performance, diarrhea index score, dietary digestibility and metagenomics of the deep rectum microbiota were evaluated.

**Results:**

The TAN had greater weight gain in the nursery phase and final weight (p<0.05) than the CONTR (394 vs 360 g/d, and 22.6 vs 21.1 kg, respectively), with these values being intermediate for the ENR+ZnO and BUT (365 and 382 g/d, and 21.3 and 22.1 kg, respectively). There was no difference between treatments for semi-liquid diarrhea (score 2), but CONTR had more cases of severe diarrhea (score 3; p<0.05) than ENR+ZnO, BUT and TAN, with 42, 18, 29, and 21 cases, respectively. The treatments had no impact on rare taxa or the relative abundances of taxonomic groups (uniformity), but the use of TAN promoted an increase in the abundances of *Brevibacillus* spp. and *Enterococcus* spp. compared to the other treatments (p<0.05).

**Conclusion:**

The use of condensed tannin from black wattle as a performance-enhancing additive was effective, with effects on performance and intestinal health, demonstrating its potential as a substitute for zinc oxide and enramycin in the diets of piglets in nursery phase.

## INTRODUCTION

Modern swine farming, even with innovative technologies, results in unavoidable stressful conditions at the time of weaning, limiting the digestion and absorption of nutrients, water, and electrolytes, in addition to impairing the protective barrier of the gastrointestinal tract [1]. Consequently, intestinal permeability increases, which favours the establishment of pathogens such as *Escherichia coli* (*E. coli*), increasing the frequency of cases of diarrhea [2].

Antibiotics have been used for decades in prophylactic conditions or as growth promoters, effectively addressing the limitations and challenges inherent in the post-weaning period by promoting improved weight gain and feed conversion ratio, and reducing diarrhea and mortality in nursery piglets [2,3]. However, their use presents significant risks, including the selection and multiplication of resistant pathogens [4], masking of subclinical conditions that lead to decreased performance, and potential risks to human health, resulting in their global banishment [3]. Similarly, zinc oxide, commonly used at doses between 2,000 and 3,000 mg/kg to reduce the symptoms and severity of post-weaning diarrhea, has proven to be highly effective. Nevertheless, its high-dose use poses an environmental risk [5], as a substantial portion of the zinc (60% to 80%) is excreted in the feces [6], leading to bans on its use at high concentrations in many countries [3].

The use of alternative additives as substitutes for antimicrobials is gaining ground within the swine industry, aiming for similar or superior results compared to antimicrobials and zinc oxide without posing risks to animals, the environment, or humans. In this context, tannins, which are naturally occurring polyphenolic compounds found in various plant species, have emerged as a promising alternative additive due to their astringent, antimicrobial, and antioxidant properties. They can be divided into two categories: hydrolysable and condensed tannins, each offering unique benefits and applications [7]. Condensed tannins, or proanthocyanidins, are polymers of flavonoids that are not susceptible to hydrolysis and are consequently poorly absorbed in the gastrointestinal tract [8]. This class of tannins is found in many vegetables, particularly in black wattle (*Acacia mearnsii*), a tree native to Australia that is among the richest sources of this compound [9].

Regarding its potential as a feed additive for production animals, tannins act as a performance enhancer by optimizing protein metabolism, increasing the absorption of amino acids, and due to factors associated with improved intestinal health [10]. Their antioxidant, anti-inflammatory and antimicrobial properties may help modulate the intestinal microbiota, reducing the incidence of diarrhea, especially in the post-weaning phase, when the animal is more vulnerable to this condition [10]. The antimicrobial effects of tannins result from distinct actions that include structural or functional changes in the bacterial membrane, damage to the physicochemical properties mainly linked to the hydrophobicity of the bacterial cell surface [11] and competition that they exert with signalling molecules of the bacterial membrane receptors involved with quorum sensing [12].

In swine, tannin use is not as popular as other alternative additives; however, Girard et al [13], when using 1% chestnut tannin extract, were successful in controlling diarrhea in piglets challenged with *E. coli* ETEC F4, and Ma et al [14], using tannin from quebracho (*Schinopsis lorentzii*) for nursery piglets, also found an improvement in the incidence of diarrhea, with positive repercussions on intestinal morphometry. In turn, Biagi et al [15], evaluating increasing doses of Brazil nut (*Castanea sativa mill*) tannin for weaner piglets, found positive effects on health and performance compared to a group that did not receive this additive.

Given the widespread use of butyrate and other acids as feed additives to replace antibiotics as growth promoters and zinc oxide [16], particularly following European restrictions on these substances—and considering the systematic use of enramycin in conjunction with zinc oxide in major pig-producing countries such as China and the USA for controlling diarrhea in piglets [17], this study explores alternative solutions. Recognizing the diverse sources and limited information available on the potential of condensed tannins for production and animal health, we hypothesized that the use of condensed tannin from black wattle (*A. mearnsii*) could effectively replace these traditional additives. Thus, our study aimed to evaluate the efficacy of black wattle extract in enhancing performance, controlling diarrhea, improving digestibility, and modulating the intestinal microbiota as compared to the established treatments of enramycin with zinc oxide and sodium butyrate.

## MATERIALS AND METHODS

### Ethics committee

All procedures performed in this study were previously reviewed and approved by the Ethics Committee for Research and Experimental Animals of Akei Animal Research; the approval reference number is 014/20.

### Animals

A total of 200 piglets, half castrated males and half females, of the PIC genetic line (Camborough X AG 337) that had an average age of 22 days, and a live weight of 6.0±0.9 kg were used.

### Experimental design and treatments

The experimental design was randomized blocks (which were formed based on the initial weights of the animals and sex), with four treatments and 10 replicates per treatment. A pen with five animals of the same sex formed the experimental unit. The animals had free access to feeds and water throughout the experimental period (22 to 64 days of age), and the nutritional program [18] was divided into four phases: pre-initial I (22 to 29 days), pre-initial II (29 to 43 days), initial I (43 to 50 days) and initial II (50 to 64 days; Table 1). The following treatments were applied within each nutritional phase: CONTR, control diet (free of growth promoting additives); ENR+ZnO, control diet with the addition of antibiotics (10 mg/kg enramycin) + 2,500 mg/kg zinc oxide in pre-initial phases I and II; BUT, control diet with the addition of 900 mg/kg sodium butyrate; TAN, control diet with the addition of condensed tannins (2,000 mg/kg).

In the TAN treatment, extract of the black wattle (*A. mearnsii*) plant was added to the rations with the product Tanfeed, which contains approximately 47.8% tannin. This served as a source of condensed tannins at a concentration of 2,000 mg/kg. The tannin dose was based on Caprarulo et al [10] review.

### Experimental management

Piglets were weighed at 0, 7, 21, 28, and 42 d of experiment individually to calculate the average daily gain (ADG). Feed intake was recorded during the experimental period for each pen every two weeks to calculate the average daily feed intake (ADFI). The ADG:ADFI (G:F) for each pen was calculated subsequently.

The diarrhea score was determined daily and classified as follows: 0, stools of normal consistency; 1, soft stools; 2, pasty stools; and 3, aqueous stools [19]. The diarrhea severity index was calculated according to the equation:


$$\text{Diarrhea severity index}=\frac{\text{sum of diarrhea scores }(2\text{: pasty and }3\text{: aqueous stools})}{\text{number of total animals evaulated that day }(42\;\text{days}\times 50\;\text{pigles})}$$

On the 21st day of the experiment, feces were collected from the deep rectum using a swab. Forty animals were sampled, and one animal from each replicate was randomly chosen. The content (approximately 2 g) was transferred to an Eppendorf tube, which was refrigerated for maintenance and immediately frozen at −80°C. The samples were subjected to metagenomic analysis using the ZR Fecal DNA MiniPrep kit from Zymo Research (No. D6010; Zymo Research, Irvine, CA, USA) to extract DNA from the samples, following the protocol recommended by the manufacturer. The extracted DNA was quantified by spectrophotometry at 260 nm (Thermo Fisher Scientific, Waltham, MA, USA). To evaluate the integrity of the extracted DNA, all samples were run by electrophoresis on a 1% agarose gel (Bio-Rad Laboratories, Hercules, CA, USA).

A segment of approximately 460 bases of the V3–V4 hypervariable region of the 16S rRNA ribosomal gene was amplified using universal primers and the following PCR conditions: 95°C for 3 min; 25 cycles of 95°C for 30 s, 55°C for 30 s, and 72°C for 30 s; followed by a step at 72°C for 5 min. The metagenomic library was constructed from these amplicons using the Nextera DNA Library Preparation Kit from Illumina (No. FC-131-1096; Illumina, San Diego, CA, USA). The amplicons were pooled and subsequently sequenced on the Illumina MiSeq sequencer (No. SY-410-1003; Illumina, USA) [20].

The sequencer readings were analysed on the quantitative insights into microbial ecology (QIIME) platform [21]. The sequences were classified into bacterial genera through the recognition of operational taxonomic units (OTUs), in this case, the homology between the sequences and those in a database. The 2017 update (SILVA 128) of the SILVA ribosomal sequence database [22] was used to compare the sequences.

At 42 days of age, during initial phase I, the animals were subjected to experimental diets containing 0.3% chromic oxide as an indigestible marker. This decision aligns with findings by Tang et al [23], who noted that intestinal barrier function is impaired at the beginning of weaning but begins to recover after two weeks. An adaptation period of at least 5 days was necessary for accurate digestibility measurements in pigs fed diets with 1.8% to 3.3% crude fiber. Previous studies indicate that fecal chromium concentrations stabilize by day 5, ensuring consistent apparent total tract digestibility (ATTD) values [24,25]. After 5 days of consumption of each of these diets, feces were collected from the animals for three consecutive days, totaling one pool of feces per pen, with a volume of approximately 500 g. Afterwards, the feces were stored at −20°C until they were dried in a forced ventilation oven at 62°C for 72 h. The samples were subjected to determination of dry matter, performed by drying in a forced air oven (Thermo Fisher Scientific, USA) at 105°C for 6 h (method 934.01); ash and crude protein, analysed using the methods 942.15 and 990.03, respectively [26]; and gross energy, determined by an automatic adiabatic oxygen pump calorimeter (Parr 1281, Automatic Energy Analyzer; Parr Instrument Company, Moline, IL, USA). The chromium content in the diets and feces was measured using an atomic absorption spectrophotometer (PerkinElmer, Waltham, MA, USA). With these data, the ATTD [27] in initial phase I was calculated using the following equation:


$$\text{ATTD}\;(\%)=100-\left[\left(\frac{\%{\text{Chromium}}_{\text{feces}}}{\%{\text{Chromium}}_{\text{feed}}}\right)\times \left(\frac{\%{\text{N}}_{\text{feces}}}{\%{\text{N}}_{\text{feed}}}\right)\times 100\right]$$

Where, %Chromium_feces_ is the percentage of the chromium indicator in the feces; %Chromium_feed_ is the percentage of the chromium indicator in the feed; %N_feces_ is the percentage of the nutrient in the feces; %N_feed_ is the percentage of the nutrient in the feed.

### Statistical analyses

In this study, the experimental design was a randomized complete block design, considering the sex (male and female) and initial weight of the piglets as blocking factors. These factors were used to classify piglets into five weight categories, aiming to ensure homogeneity within each block. The fixed effects in the model included the treatment itself, the block defined by sex and weight category, and the interaction between treatment and block. Random effects were accounted for by the variability between pens within each block, recognizing that individual pen characteristics might influence outcomes. The statistical model was:


$${\text{Y}}_{\text{ijkl}}=\mu +\tau_{\text{i}}+\beta_{\text{j}}+{\left(\tau \beta \right)}_{\text{ij}}+\gamma \text{k}(\text{j})+{ɛ}_{\text{ijkl}}$$

In the statistical model employed, Y_ijkl_ denotes the observed measurement, where μ represents the overall mean. The fixed effects are captured by τ_i_, which represents the effect of the ith treatment, and β_j_, which denotes the effect of the jth block, such as sex and weight categories. The interaction between these treatments and blocks is expressed as (τβ)_ij_. The model also includes random effects, denoted by γk(j), which account for the variability among pens within each block. Lastly, ε_ijkl_ captures the residual error for the lth observation in the kth pen of the jth block under the ith treatment. Statistical analyses were conducted using R statistical software, version 3.5.0. Means were compared using Tukey's test, and differences were considered statistically significant at a p-value of ≤0.05. Trends were noted where p-values ranged between 0.05 and 0.10. Additionally, Chi-square tests were used to compare the frequency of diarrhea across treatments, applying the same criteria for significance.

The differences in the alpha diversity observed and relative abundance between the groups were estimated by the Kruskal-Wallis test, and the differences between the different treatments were estimated by post-hoc Dunn test. The effect of treatments on beta diversity was assessed between groups using permutational multivariate analysis of variance [28], with multiple comparisons corrected by the Bonferroni test.

## RESULTS

The phase-by-phase zootechnical performance throughout the experimental period is shown in Table 2. There were no significant differences between treatments during the pre-initial phase I (22 to 29 days of age). However, during the pre-initial phase II (29 to 43 days of age), the treatment with TAN showed significant improvements over the CONTR group in several zootechnical parameters: ADFI (p = 0.041), ADG (p = 0.006), G:F (p = 0.007), and body weight (p = 0.036). Additionally, there were trends indicating better body weight at the end of the initial phase I (p = 0.067) and at the end of the experiment (p = 0.085), as well as improved ADG over the entire nursery phase (p = 0.067).

Relative to the other treatments (ENR+ZnO and BUT), the results were intermediate and similar to those of the other groups (CONTR and TAN), mainly for ADG considering the total test period and final weight, with some specific advantages for the ADFI (p = 0.041) and ADG (p = 0.006) compared to the CONTR in pre-initial phase II.

Table 3 shows the cases and severity of diarrhea throughout the study period, with significant reductions in the most severe score and in the total score (scores 2 plus 3) for all treatments that received additives compared to the CONTR group (p<0.001).

**Figure 1:**
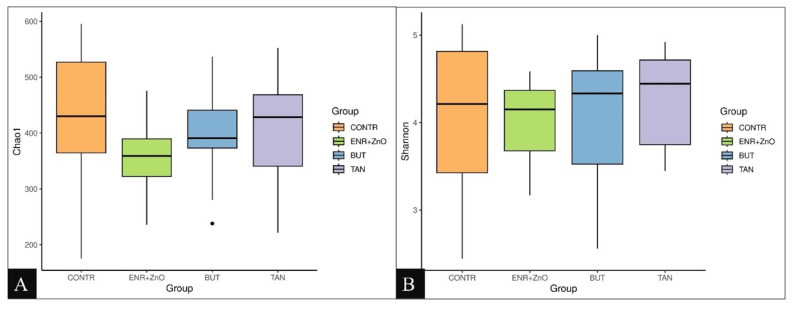
Chao1’s alpha diversity index (A). Shannon’s alpha diversity index (B). Non-parametric Kruskal-Wallis test and post-hoc Dunn test were performed (p>0.05). CONTR, control diet (free of growth promoting additives); ENR+ZnO, control diet with the addition of antibiotics (10 mg/kg enramycin) + 2,500 mg/kg zinc oxide in pre-initial phases I and II; BUT, control diet with the addition of 900 mg/kg sodium butyrate; TAN, control diet with the addition of 2,000 mg/kg condensed tannins. Chao1's alpha diversity index measures the richness of the microbial community, estimating the total number of species present in a sample. Shannon’s alpha diversity index quantifies the diversity within microbial communities, considering both abundance and evenness of the species present.

The evaluation of the alpha diversity of the microbiota of the feces from the deep rectum, demonstrated by the Chao1 bias-corrected index (Figure 1A) and Shannon entropy (Figure 1B), indicated that the treatments with additives did not affect either the number of different species in each group (richness), including the rare species, or the relative abundance of each taxonomic group present in the samples (uniformity).

**Figure 2:**
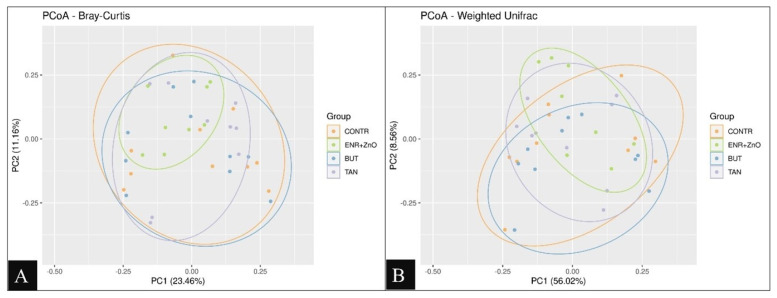
Weighted Unifrac Beta Diversity distance measures (A). Bray-Curtis Beta Diversity dissimilarity measures (B). Ellipses were automatically plotted with ggforce R package. CONTR, control diet (free of growth promoting additives); ENR+ZnO, control diet with the addition of antibiotics (10 mg/kg enramycin) + 2,500 mg/kg zinc oxide in pre-initial phases I and II; BUT, control diet with the addition of 900 mg/kg sodium butyrate; TAN, control diet with the addition of 2,000 mg/kg condensed tannins. Weighted UniFrac beta diversity distance measures: This index assesses the similarity between microbial communities, taking into account both the phylogenetic distances and the relative abundances of different species present. Bray-Curtis beta diversity dissimilarity measures: This measure quantifies the dissimilarity between two microbial communities based on the counts of species they contain. Unlike UniFrac, Bray-Curtis does not consider phylogenetic relationships and is purely a measure of species abundance and composition differences.

When analyzing the beta diversity (Figure 2A and 2B), there was no formation of sample clusters according to treatment when considering the phylogenetic dissimilarity and abundance of the identified taxa, confirming the absence of significant differences between treatments.

**Figure 3:**
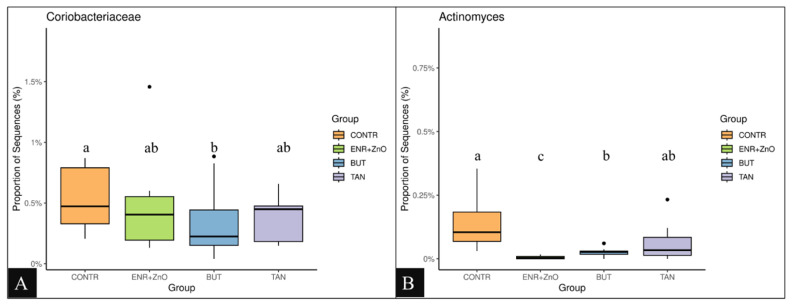
Proportion of sequences from *Coriobacteriaceae* in tested groups (A). Proportion of sequences from *Actinomyces* in tested groups (B). Non-parametric Kruskal-Wallis test and post-hoc Dunn test were performed (p<0.05). CONTR, control diet (free of growth promoting additives); ENR+ZnO, control diet with the addition of antibiotics (10 mg/kg enramycin) + 2,500 mg/kg zinc oxide in pre-initial phases I and II; BUT, control diet with the addition of 900 mg/kg sodium butyrate; TAN, control diet with the addition of 2,000 mg/kg condensed tannins.

**Figure 4:**
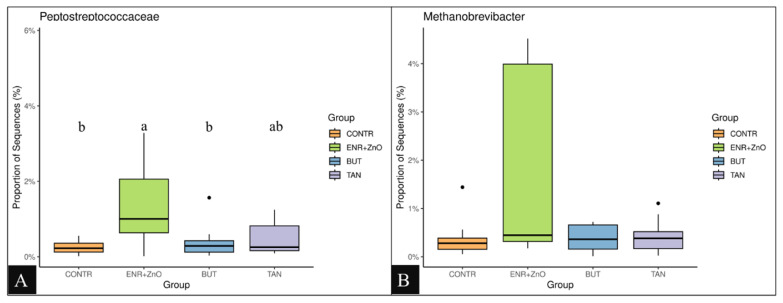
Proportion of sequences from *Peptostreptococcaceae* in tested groups (A). Proportion of sequences from *Methanobrevibacter* in tested groups (B). Non-parametric Kruskal-Wallis test and post-hoc Dunn test were performed (p<0.05). CONTR, control diet (free of growth promoting additives); ENR+ZnO, control diet with the addition of antibiotics (10 mg/kg enramycin) + 2,500 mg/kg zinc oxide in pre-initial phases I and II; BUT, control diet with the addition of 900 mg/kg sodium butyrate; TAN, control diet with the addition of 2,000 mg/kg condensed tannins.

**Figure 5:**
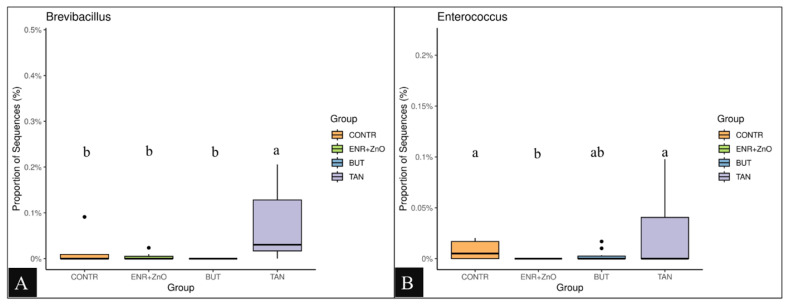
Proportion of sequences from *Brevibacillus* in tested groups (A). Proportion of sequences from *Enterococcus* in tested groups (B). Non-parametric Kruskal-Wallis test and post-hoc Dunn test were performed (p<0.05). CONTR, control diet (free of growth promoting additives); ENR+ZnO, control diet with the addition of antibiotics (10 mg/kg enramycin) + 2,500 mg/kg zinc oxide in pre-initial phases I and II; BUT, control diet with the addition of 900 mg/kg sodium butyrate; TAN, control diet with the addition of 2,000 mg/kg condensed tannins.

For the distribution of each experimental group, there was no prevalence of any representative of the taxonomic groups (classes, orders, families, and quantified genera) among the treatments; however, greater abundances were found at the family and genus levels for the CONTR and ENR+ZnO. As shown in Figure 3, the family *Coriobacteriaceae* (Figure 3A) and the genus *Actinomyces* (Figure 3B) were the most abundant taxa in the treatment that did not receive any additive promoting intestinal performance and health (CONTR). Similarly, the abundance of *Actinomyces* was higher in the CONTR group compared to the ENR+ZnO and BUT groups (p<0.001). As shown in Figure 4A, the family *Peptostreptococcaceae* was more abundant in the animals that received ENR+ZnO compared to the CONTR and BUT groups (p = 0.046). The abundance of the genus *Methanobrevibacter* was similar across all treatments (Figure 4B). In contrast, Figure 5A shows that *Brevibacillus* spp. were more abundant in the TAN group compared to the CONTR, ENR+ZnO, and BUT groups (p<0.001). Meanwhile, Figure 5B indicates that *Enterococcus* spp. showed no difference in abundance between the TAN, BUT, and CONTR groups, but the abundance was decreased in the ENR+ZnO group (p = 0.030).

Table 4 shows the digestibility coefficients of the dry matter, crude protein, mineral matter and gross energy of the experimental diets. No differences were observed between TAN and the other treatments. Only the ENR+ZnO showed an improvement in the index (p = 0.011) of mineral matter digestibility compared to the CONTR and BUT treatments, although ENR+ZnO did not differ from the group that received TAN.

## DISCUSSION

The benefits of the use of condensed tannin of black wattle (*A. mearnsii*) for growth performance of pigs indicate that this active ingredient was effective for the parameters investigated, as demonstrated by the results of this study (Table 2). The findings indicate that, depending on the characteristics of the molecule and dose used, the risk that tannins may be an inhibitor of feed consumption due to its rancid odour and bitter taste, in addition to constituting an antinutritional factor, is not valid [29]. Such ideas, which led to a negative perception about the molecule, result from its use at high concentrations and from tannins with different molecular bases [30].

Therefore, the results of this study confirm that to achieve the benefits we observed, the chemical structure, origin and dosage of the tannin used must be taken into account. Working with 1% chestnut tannin extract for prevention in piglets challenged or not by *E. coli* F4, Girard et al [13] observed no reduction in the feed intake of the animals, showing that there was no antinutritional effect due to the tannins. These results were also observed in our study.

The positive responses obtained with the condensed black wattle (*A. mearnsii*) tannins (TAN) are also due to the good feed intake among pigs in the group given this additive, which is outstanding relative to the feed intake in other treatments, with significant advantages compared to the CONTR group in the phase between 29 and 44 days of age (Table 2). In the study by Ma et al [14], the inclusion of up to 0.3% quebracho tannin in piglet feed did not affect feed intake or weight gain. These results, which differ from those obtained for TAN, may be related to the amount and type of tannin used, age of the animals, basal diet ingredients and state of hygiene and storage.

Regarding feed consumption, a meta-analytic study suggests that low concentrations of tannins from various sources can enhance this zootechnical parameter, positively impacting performance in weaned piglets [31]. This benefit is believed to stem from the ability of tannins to form complexes with macromolecules and proline-rich protein compounds, which remain stable across different pH levels in the digestive tract. This increases the efficiency of nutrient utilization from the diet and mitigates the negative impact on palatability and feed consumption [32,33].

The positive results observed in the zootechnical performance of pigs treated with TAN, as detailed in Table 2, are particularly noteworthy because they are comparable to those of ENR+ZnO and BUT, which are both recognized and widely used additives. These effects can be attributed to the beneficial impact of tannins on protein metabolism, which enhances amino acid absorption and improves intestinal health, aligning with the findings of several studies [29,34]. In addition, the antioxidant, anti-inflammatory and antimicrobial properties of tannins should be considered, as they may help in the modulation of intestinal microbiota, reducing the incidence of diarrhea, especially in the post-weaning phase, when animals are more vulnerable to this condition [10].

Our results are similar to those obtained by Biagi et al [15], who added 1.13, 2.25, and 4.5 g of nut (*Castanea sativa* mill) tannin per kilogram of feed for weaner piglets. Compared to a group that did not receive this additive, an improvement in feed efficiency, a reduction in caecal ammonia concentrations and a promotion of health status were observed; the authors attributed these effects to the antimicrobial properties of the tannins and the inhibitory effects they have on bacterial toxins. In our study, the concentration of ammonia in the cecum was not recorded, but no difference was observed in the abundance of the *Methanobrevibacter* family, the main dominant phylotype of methanogenic bacteria in swine [35].

Our results demonstrate that TAN is as effective as additives containing ENR+ZnO and BUT in terms of zootechnical performance, showing similar effects on performance parameters. Although enramycin helps modulate intestinal bacteria against gram-positive bacteria and zinc oxide acts as an enzyme cofactor that aids in diarrhea prevention [36], these additives did not achieve higher performance indices than those obtained with TAN. Comparing the results of the group that received TAN with the group treated with BUT, a product widely used as an alternative to growth-promoting antibiotics, whose actions include improving performance and intestinal health and controlling diarrhea [37], a similar pattern of results was verified.

All the treatments that received additives (ENR+ZnO, BUT, and TAN) had a reduced incidence and severity of diarrhea (Table 3). The results observed for tannins were similar to those observed by Girard et al [13], who studied piglets that received an oral suspension of ETEC F4 and noted that the addition of 1% chestnut tannin extract reduced the mean faecal score, the percentage of piglets with diarrhea and the duration of diarrhea. However, it is not sufficient to reduce the shedding of *E. coli*. The authors also emphasize that increasing the dose of tannin extract may improve its efficiency in controlling diarrheal conditions, but attention should be given to the possible antinutritional effects on protein digestion or on changes in palatability of the feed due to the use of higher doses.

Condensed tannins, also known as proanthocyanidins, are flavonoid polymers, compounds known to have biological activities, including anti-inflammatory, anti-carcinogenic, antiviral and antibacterial activities; these effects are determined by the antioxidant properties of the molecules [38]. These qualities may explain the positive effects we observed on the control of diarrhea with the use of this molecule.

According to Ma et al [14], who used tannins from quebracho (*Schinopsis lorentzii*) for nursery pigs, supplementation with 0.3% of this commercial extract reduced the incidence of diarrhea among piglets weaned early, promoting increases in the jejunal villi height and crypt depth and a reduction in the colonic mucosa. The improvement in the diarrhea index was associated with low neutrophil levels, which are directly related to intestinal homeostasis and diseases, representing a key component of the innate response during an inflammatory reaction [39].

In the control of diarrhea, it should be noted that phenolic compounds not only have an antimicrobial action due to the structural or functional damage they confer to bacterial cell membranes [11], but also can affect the physicochemical properties of species by affecting the bacterial cell surface, mainly due to its hydrophobicity, in addition to altering the electron acceptors and polar and nonpolar components of bacteria [40]. Another antimicrobial effect of condensed tannins is achieved through competition with signalling molecules, such as acyl homoserine lactone, which bind to receptors on the bacterial membrane involved in quorum. This condition is currently of great importance, as this process (quorum sensing) does not determine bacterial resistance [12,41].

Regarding the metagenomic evaluation, the absence of differences in the alpha and beta diversities between the treatments shows that the additives did not results in a sufficient stimulus to modulate these traits. However, when analysing taxa with greater abundances, *Coriobacteriaceae* and *Actinomyces* were respectively the most abundant family and genus in the CONTR treatment (Figure 3), being the first an opportunistic pathogen related to post-infection conditions [42], and the second positively correlated with the concentration of putrescine [43], an amine related to intestinal disorders.

The abundances of the family *Peptostreptococcaceae* and the genus *Methanobrevibacter* were higher in the ENR+ZnO compared with CONTR and BUT, but it was similar to TAN (Figure 4A and 4B, respectively). The *Peptostreptococcaceae* family is associated with weight gain in pigs [44] and is observed in greater abundance in animals with better intestinal health [45], even in cases with lower feed intake. However, this action was not observed to the TAN treatment.

In turn, the abundance of the *Methanobrevibacter* family was similar between treatments, not exerting any favors regarding the modulation of ammonia production. These findings are different from Min et al [46], who observed a proportional increase in this family according to the concentration of tannins in the diet. It can be attributed that the diversity of the tannin source and the concentration used in our work did not favor the greater abundance of *Methanobrevibacter*.

Regarding the TAN treatment, it influenced specific modulations, notably increasing the abundance of the genus *Brevibacillus* spp. as shown in Figure 5A, compared to the other treatments. However, the abundance of *Enterococcus* spp. in the TAN group did not differ from the BUT and CONTR groups, as illustrated in Figure 5B. The genus *Brevibacillus* has the ability to biotransform tannins of plant origin [48] in addition to having a probiotic effect [47], observed especially in some species of the genus (*B. laterosporus* and *B. brevis*) [48], as well as a recognized inhibitory effect against *L. monocytogenes* [49]. Additionally, *B. brevis* is closely related to improved feed conversion and performance of nursery piglets [50].

The genus *Enterococcus* was also more abundant in the TAN treatment. In this context, within some species, such as *E. cecorum* and *E. durans*, *Enterococcus* has been highlighted for the advantages it has in the control of diarrheal conditions in animals [51]. Additionally, the species *E. faecium* has a particular probiotic effect that is related to the production of organic acids [52].

In contrast, tannins from Brazil nut (*Castanea sativa mill*) showed a positive effect, determined by the tendency to increase viable *lactobacilli* in the jejunum; however, there was no influence on caecal *lactobacilli* [15]. At the same time, the caecal coliform count showed a tendency to increase, while clostridia and enterococci were not affected by supplementation [15].

Tannins are a group of water-soluble polyphenols, categorized as hydrolyzable and non-hydrolyzable (condensed), each possessing distinct antibacterial activities [53]. Their effects also depend on their composition, which is influenced by the plant of origin and the extraction method of the substance [54]. For these reasons, the modulation of lactic acid bacteria, especially lactobacilli, may vary between studies, leading to inconsistent responses across different research. The modulation of the intestinal microbiota is a function of condensed tannins, resulting from a set of properties that directly or indirectly determine the condition, highlighting the antioxidant, anti-inflammatory and antimicrobial activities they possess [10].

Condensed tannin (*A. mearnsii*) did not affect the ATTD of dry matter, crude protein, mineral matter and gross energy of the diets, behaving similarly to the other treatments (Table 4). The notion that tannins are anti-nutritional substances due to their ability to precipitate proteins, inhibit digestive enzymes, and reduce nutrient utilization was not supported by this evaluation [55]. It confirms that the dietary concentration and molecular characteristics of tannins must be considered. Consistent with this, Girard et al [13] reported no performance detriment or antinutritional effects from using tannins as an additive in piglet diets. However, the observed enhancement in mineral digestibility for the ENR+ZnO diet compared to the CONTR and BUT diets aligns with findings by Mille et al [56]. In their study using pharmacological levels of zinc for weaned piglets, Mille et al [56] did not observe improvements in the digestibility of other nutrients, yet they noted enhanced rates of mineral retention. This improvement can be attributed to the role of zinc oxide in enhancing digestion by stimulating pancreatic and intestinal enzymatic activities [57], as well as by improving the morphology of the small intestine [58]. Furthermore, the addition of enramycin to zinc oxide appears to enhance this digestive benefit, contributing to the activity of digestive enzymes and reducing damage to the intestinal mucosa structure [59].

The use of tannin as an additive has not compromised the digestibility of amino acids and proteins, as evidenced by other studies [60,61]. This supports the notion that tannin's ability to bind to proteins does not necessarily exert negative effects. Instead, the formation of tannin-protein complexes may protect proteins, carbohydrates, and lipids from oxidative damage during digestion [62]. These results confirm the suitability of using condensed tannins from black wattle (*A. mearnsii*) at the dose applied as a performance-enhancing additive without risk of negatively influencing the digestibility of dietary nutrients.

## CONCLUSION

The condensed tannin from the extract of black wattle (*A. mearnsii*), as an additive for piglets in the nursery phase, was effective in controlling diarrheal conditions, did not affect the digestibility of the nutrients in the diet and promoted specific modulations of the intestinal microbiota, with positive results on zootechnical performance. The results demonstrate that condensed black wattle tannin are an effective substitute for zinc oxide associated with enramycin and led to results similar to those achieved in diets that used sodium butyrate.

## Figures and Tables

**Table 1:** Composition and nutritional values of the rations used in the experimental procedures for the pre-initial I and II and initial I and II phases (as-is basis)

Items	Phases			

	Pre-initial I	Pre-initial II	Initial I	Initial II
Ingredients (%)				
Corn grain	44.503	42.045	51.775	60.630
Soybean meal	13.161	22.048	26.352	30.838
Pregelatinized corn flour	10.000	10.000	5.000	0.000
Whey powder	14.000	12.000	7.000	0.000
Milk powder	10.000	5.000	2.500	0.000
Blood plasma	5.000	4.000	2.500	0.000
Soybean oil	0.208	1.075	1.519	4.199
Dicalcium phosphate	1.110	1.073	1.452	1.833
Limestone	0.280	0.506	0.547	0.765
L-lysine	0.543	0.316	0.365	0.397
L-threonine	0.256	0.121	0.150	0.157
DL-methonine	0.360	0.214	0.185	0.156
L-valine	0.039	0.000	0.000	0.033
L-tryptophan	0.051	0.012	0.012	0.024
Adsorvent	0.150	0.150	0.150	0.150
Salt	0.032	0.132	0.233	0.568
Choline chloride	0.047	0.047	0.000	0.000
Antioxidant	0.010	0.010	0.010	0.010
Vitamin premix^1)^	0.150	0.150	0.150	0.150
Mineral premix^2)^	0.100	0.100	0.100	0.100
Total	100.00	100.00	100.00	100.00
Nutrients				
Metabolizable energy (kcal/kg)	3,500	3,400	3,350	3,350
Crude protein (%)	18.500	20.045	20.000	19.450
Crude fat (%)	4.837	4.383	4.461	6.640
Crude fibre (%)	1.801	2.355	2.682	3.053
Calcium (%)	0.640	0.697	0.750	0.872
Available phosphorus (%)	0.440	0.401	0.420	0.431
SID lysine (%)	1.440	1.334	1.300	1.206
SID methionine + cysteine (%)	0.927	0.814	0.770	0.687
SID threonine (%)	0.940	0.855	0.840	0.784
SID tryptophan (%)	0.250	0.240	0.230	0.229
SID valine (%)	0.857	0.892	0.813	0.832
SID isoleucine (%)	0.696	0.775	0.778	0.749
Sodium (%)	0.320	0.300	0.250	0.250
Chlorine (%)	0.380	0.346	0.337	0.366

SID, standardized ileal digestibility.

1) Rovimix (DSM, Heerlen, Netherlands): levels per kg of vitamin premix product: 6,000 IU of vitamin A as vitamin A acetate; 1,500 IU of vitamin D_3_; 15,000 mg of vitamin E; 1,500 mg of vitamin K_3_ as menadione sodium bisulfate; 1,350 mg of vitamin B_1_ as thiamine mononitrate; 4,000 mg of vitamin B_2_ as riboflavin; 2,000 mg of vitamin B_6_ as pyridoxine hydrochloride; 20 mg of vitamin B_12_ as cyanocobalamin; 20,000 mg of niacin as nicotinic acid; 9,350 mg of pantothenic acid as calcium pantothenate; 600 mg of folic acid; 80 mg of biotin; 300 mg of selenium as sodium selenite.

2) Oligomix (DSM, Heerlen, Netherlands): levels per kg of mineral premix product: 100 mg of iron as ferrous sulfate; 10 mg of copper as copper sulfate; 40 g of manganese as manganous oxide; 1,000 mg of cobalt as cobalt sulphate; 100 mg of zinc as zinc oxide; 1,500 mg of iodine as calcium iodine.

**Table 2:** Means (±SEM) of average daily feed intake (ADFI), average daily gain (ADG), gain-to-feed ratio (G:F) and initial body weight (IBW) and final body weight (FBW), according to the experimental phases and throughout the study period

Items	Treatments^1)^				SEM	p-value

	CONTR	ENR+ZnO	BUT	TAN		
Pre-Initial I (d 22–29)						
IBW (kg)	6.0	6.0	6.0	6.0	0.14	0.995
ADFI (g/d)	164	163	163	160.0	4.60	0.988
ADG (g/d)	119	133	121	114	5.05	0.630
G:F	0.727	0.806	0.728	0.711	0.018	0.292
FBW (kg)	6.8	6.9	6.8	6.7	0.14	0.618
Pre-Initial II (d 29–43)						
ADFI (g/d)	389^b^	452^a^	449^a^	445^a^	9.14	0.041
ADG (g/d)	253^b^	304^a^	296^ab^	330^a^	8.35	0.006
G:F	0.640^b^	0.673^ab^	0.659^b^	0.746^a^	0.011	0.007
FBW (kg)	10.4^b^	11.2^a^	11.0^ab^	11.4^a^	0.19	0.036
Initial I (d 43–50)						
ADFI (g/d)	593^b^	625^ab^	682^a^	670^ab^	12.06	0.082
ADG (g/d)	349	350	405	395	10.44	0.220
G:F	0.532	0.626	0.599	0.635	0.019	0.658
FBW (kg)	12.8^b^	13.7^ab^	13.8^ab^	14.2^a^	0.26	0.067
Initial II (d 50–64)						
ADFI (g/d)	898	873	946	951	14.64	0.159
ADG (g/d)	595	548	588	599	10.48	0.328
G:F	0.665	0.627	0.622	0.630	0.007	0.256
FBW (kg)	21.1^b^	21.3^ab^	22.1^ab^	22.6^a^	0.33	0.085
Total (d 22–64)						
ADFI (g/d)	555	573	606	603	9.51	0.197
ADG (g/d)	360^b^	365^ab^	382^ab^	394^a^	8.35	0.067
G:F	0.650	0.637	0.632	0.654	0.004	0.324

SEM, standard error of mean.

1) CONTR, control diet (free of growth promoting additives); ENR+ZnO, control diet with the addition of antibiotics (10 mg/kg enramycin) + 2,500 mg/kg zinc oxide in pre-initial phases I and II; BUT, control diet with the addition of 900 mg/kg sodium butyrate; TAN, control diet with the addition of 2,000 mg/kg condensed tannins.

a,b Means with different letters on the same line are different (p<0.05) according to the Tukey test.

**Table 3:** Incidences and severity of diarrhea, according to the treatments

Items	Treatments^1)^				p-value

	CONTR	ENR+ZnO	BUT	TAN	
Score 2 (n)	6	1	1	5	0.077
Score 3 (n)	42^b^	18^a^	29^a^	21^a^	<0.001
Total (n)	48^b^	19^a^	30^a^	26^a^	<0.001
Diarrhea severity index^2)^	0.022	0.009	0.014	0.012	-

1) CONTR, control diet (free of growth promoting additives); ENR+ZnO, control diet with the addition of antibiotics (10 mg/kg enramycin) + 2,500 mg/kg zinc oxide in pre-initial phases I and II; BUT, control diet with the addition of 900 mg/kg sodium butyrate; TAN, control diet with the addition of 2,000 mg/kg condensed tannins.

2) The diarrhea score was determined daily and classified as follows: 2 = pasty stools; and 3 = aqueous stools. Diarrhea severity index = (sum of diarrhea scores (2: pasty and 3: aqueous stools ))/(number of total animals evaluated that day). n = numbers of cases.

a,b Means with different letters on the same line are different (p<0.05) according to the Qui-square test.

**Table 4:** Means of the apparent total tract digestibility (%) of dry matter (DM), crude protein (CP), mineral matter (MM) and gross energy (GE) of initial phase I according to the experimental treatments

Items	Treatments^1)^				SEM	p-value

	CONTR	ENR+ZnO	BUT	TAN		
DM	78.80	75.72	77.16	77.54	0.66	0.352
CP	70.34	65.65	66.34	67.69	1.00	0.422
MM	39.13^b^	49.89^a^	37.97^b^	42.31^ab^	1.49	0.011
GE	78.00	73.82	74.09	76.31	0.82	0.220

SEM, standard error of mean.

1) CONTR, control diet (free of growth promoting additives); ENR+ZnO, control diet with the addition of antibiotics (10 mg/kg enramycin) + 2,500 mg/kg zinc oxide in pre-initial phases I and II; BUT, control diet with the addition of 900 mg/kg sodium butyrate; TAN, control diet with the addition of 2,000 mg/kg condensed tannins.

a,b Means with different letters on the same line are different (p<0.05) according to the Tukey test.
